# Exposure to Diesel Exhaust Particle Extracts (DEPe) Impairs Some Polarization Markers and Functions of Human Macrophages through Activation of AhR and Nrf2

**DOI:** 10.1371/journal.pone.0116560

**Published:** 2015-02-24

**Authors:** Marie Jaguin, Olivier Fardel, Valérie Lecureur

**Affiliations:** 1 UMR INSERM U1085, Institut de Recherche sur la Santé, l’Environnement et le Travail (IRSET), Université de Rennes 1, 2 avenue du Pr Léon Bernard, 35043, Rennes, France; 2 Pôle Biologie, Centre Hospitalier Universitaire (CHU) Rennes, 2 rue Henri Le Guilloux, 35033, Rennes, France; McGill University, CANADA

## Abstract

Macrophages (MΦ), well-known to play an important role in immune response, also respond to environmental toxic chemicals such as diesel exhaust particles (DEP). Potential effects of DEPs towards MΦ polarization, a key hall-mark of MΦ physiology, remain however poorly documented. This study was therefore designed to evaluate the effects of a reference DEP extract (DEPe) on human MΦ polarization. Human blood monocytes-derived MΦ were incubated with IFNγ+LPS or IL-4 to obtain M1 and M2 subtypes, respectively; a 24 h exposure of polarizing MΦ to 10 μg/ml DEPe was found to impair expression of some macrophagic M1 and M2 markers, without however overall inhibition of M1 and M2 polarization processes. Notably, DEPe treatment increased the secretion of the M1 marker IL-8 and the M2 marker IL-10 in both MΦ subtypes, whereas it reduced lipopolysaccharide-induced IL-6 and IL-12p40 secretion in M1 MΦ. In M2 MΦ, DEPe exposure led to a reduction of CD200R expression and of CCL17, CCL18 and CCL22 secretion, associated with a lower chemotaxis of CCR4-positive cells. DEPe activated the Nrf2 and AhR pathways and induced expression of their reference target genes such as Hmox-1 and cytochrome P-4501B1 in M1 and M2 MΦ. Nrf2 or AhR silencing through RNA interference prevented DEPe-related down-regulation of IL-6. AhR silencing also inhibited the down-secretion of IL-12p40 and CCL18 in M1- and M2-DEPe-exposed MΦ, respectively. DEPs are therefore likely to alter expression of some M1 and M2 markers in an AhR- and Nrf2-dependent manner; such regulations may contribute to deleterious immune effects of atmospheric DEP.

## Introduction

Previous epidemiological studies have indicated that exposure to ambient particulate matter (PM) is linked to increase in mortality and morbidity related to cardio-pulmonary diseases [1]. Moreover, urban air pollution may contribute to exacerbations of asthma and to allergic airway diseases [2], but also to progression of atherosclerosis in both animal experimental models and humans [3]. Such adverse effects are more closely associated to PM with a diameter less than 2.5 μm (PM2.5) such as diesel exhaust particulates (DEP) containing adsorbed organic compounds, and to their ability to increase the release of pro-inflammatory mediators by epithelial, endothelial cells and immune cells, leading to inflammation.

Monocytes and macrophages (MΦ) play a key role in innate and adaptive immunity and inflammation. After release from bone marrow to blood, monocytes are recruited to tissues where, according to the nature of environmental signals, they can develop into myeloid dendritic cells or various forms of MΦ. The “classically activated MΦ” or M1 type generated by IFNγ followed by LPS stimulation produce high pro-inflammatory cytokines such as TNFα and IL-12/IL-23 and play a role in tissue destruction [4]. In contrast, IL-4- or IL-13-stimulated MΦ’s, so-called M2 MΦ or “alternative activated MΦ”, express membrane receptors such as scavenger receptors and mannose receptor CD206, fail to produce IL-12 cytokine but they release anti-inflammatory cytokines like IL-10 and IL-1RA and some Th2 chemokines such as CCL17 (TARC), CCL18 (PARC) and CCL22 (MDC); such M2 MΦ are involved in tissue remodelling and wound repair [4–5]. Overall, type M1 MΦ support the Th1 response whereas type M2 MΦ’s support of Th2 response.

Interestingly, DEP have been shown to up-regulate both pro-inflammatory mediators and antioxidant enzymes in various cells, including MΦ, which appear as a key cell type targeted by DEP [6–7]. DEP have also immunosuppressive effects through reducing cytokine release, notably by alveolar MΦ [8–10]. Mechanisms involved in DEP effects towards MΦ are however poorly understood. Two main families of compounds adsorbed on DEP, polycyclic aromatic hydrocarbons (PAH) and quinones, are likely to contribute to them, at least in part. Mechanistically, PAHs bind to the cytosolic aryl hydrocarbon receptor (AhR), which then migrates to the nucleus where the ligand/AhR complex heterodimerizes with the aryl hydrocarbon receptor nuclear translocator (ARNT) protein. This activated receptor-ligand complex next interacts with the xenobiotic-response element (XRE) in the promoter regions of target genes such as the phase I metabolizing enzymes cytochrome (CYP) 1A1 and 1B1. DEP extracts (DEPe) have been shown to activate AhR and to increase pro-inflammatory cytokine expression in the human monocytic U937 cell line [6]; they also induce intracellular ROS generation through CYP system in MΦ and human airway epithelial cells [11–12]. Moreover, AhR is involved in cytokine expression [13] and immune regulation [14]. The detoxification of quinones found in DEP requires the phase II enzyme NADPH-quinone oxidoreductase (NQO-1), a typical NF-E2-related factor 2 (Nrf2) target gene. In normal situations, Nrf2 protein action is repressed due to its interaction with the Keap1 protein, which results in proteosomal Nrf2 degradation. Upon oxidative and/or electrophilic stress, Nrf2 dissociates from Keap1, translocates into the nucleus, dimerizes with a small Maf protein and then binds to an antioxidant response element (ARE) found in the promoter regions of Nrf2 target genes such as antioxidant and phase II detoxification enzymes. DEPe has thus been shown to up-regulate expression of heme oxygenase-1 (HO-1) and some detoxification enzymes via ARE, in order to protect against pro-inflammatory effects of particulate pollutants in the murine macrophagic RAW 264.7 cell line [15–16]. Moreover, emerging evidence suggests that Nrf2-regulated genes may control inflammation and immune tolerance [17]. Thus, Nrf2-deficient mice exhibit more susceptibility to airway inflammation and emphysema after DEP inhalation and cigarette smoke, respectively [18–20]; moreover, Nrf2-deficient dendritic cells exposed to PM secrete more pro-inflammatory mediators than the wild-type cells [21], strongly suggesting a protective health effect of Nrf2 against air pollutants.

As DEP have been shown to impair some differentiation programs of the monocyte/ MΦ cell lineage, including dendritic cell maturation [22–24] and human monocyte differentiation and function [25], and owing to the fact that polarization is a key feature of MΦ physiology, we examined the hypothesis that DEP extract (DEPe) may alter agonist-induced human MΦ polarization process in the present study. Our results indicate that exposure to DEPe impaired expression of some macrophagic M1 and M2 markers, in an AhR- and Nrf2-dependent manner.

## Materials and Methods

### Chemicals and reagents

Human recombinant IL-4 and IFNγ were purchased from Peprotech (Neuilly sur Seine, France) whereas human recombinant M-CSF was obtained from Miltenyi Biotec SAS (Paris, France). Lipopolysaccharide (LPS) from E. Coli (serotype:055:B5), phorbol-12-myristate-13-acetate (PMA), fluorescein isothiocyanate (FITC)-dextran, tert-butylhydroquinone (tBHQ), dimethylsulfoxyde (DMSO), 3-(4,5-dimethylthiazol-2-yl)-2,5-diphenyltetrazolium (MTT) and benzo(a)pyrene (B(a)P) were purchased from Sigma–Aldrich (St Louis, MO) whereas 2,3,7,8-tetrachlorodibenzo-*p*-dioxin (TCDD) was obtained from Cambridge Isotope Laboratories (Cambridge, MA). Standard Reference Material 1975 (SRM1975), corresponding to DEPe resulting from dichloromethane-based extraction of DEP, was purchased from the National Institute of Standards and Technology (Gaithersburg, USA); dichloromethane was evaporated under nitrogen gas and the residue was dissolved in dimethyl sulfoxide (DMSO) for cell exposure to obtain a stock solution at 10 mg/ml. Final concentration of solvent DMSO in culture medium did not exceed 0.2% (v/v); control cultures received the same dose of solvent as treated counterparts.

### Polarization and exposure of human primary MΦ

Monocytes were purified from peripheral blood mononuclear cells obtained, as previously described [26]. Blood buffy coats of healthy subjects were provided by “Etablissement Français du sang” (EFS) after their written consent to use their blood sample for research. EFS is the Blood national french agency which has the autorization to supply blood sample from healthy subjects (French law No 93–5 of January 4th, 1993) whithout requirement of an ethic committee. Monocytes were then differentiated into MΦ by treatement by M-CSF (50 ng/ml) for 6 days in RPMI 1640 medium (Gibco) supplemented with 2 mM glutamine, 10% decomplemented fetal calf serum (FCS), 100 IU/ml penicillin and 100 μg/ml streptomycin. To study DEPe effect during MΦ polarization, MΦ (1x10^5^ cells/cm^2^) were next exposed for 24 h to a fresh medium supplemented with 5% FCS and containing IFNγ (20 ng/ml) (for getting type M1 MΦ) or M-CSF (10 ng/ml) + IL-4 (20 ng/ml) (for getting type M2 MΦ) [27], in absence or presence of DEPe. To study DEPe effect on already polarized MΦ, M1 or M2 MΦ, previously generated as described above, were exposed to DEPe for additional 24 h. The experiments were done in accordance with the World Medical Association declaration of Helsinki (1997) [28].

### Cell viability

Cytotoxic effect of DEP treatment toward primary human MΦ was assessed using the 3-(4–5-dimethylthiazol-2-yl)- 2,5-diphenyltetrazolium bromide (MTT) colorimetric assay. Briefly, 4-days differentiated MΦ were seeded in 96-well plates at 1x10^5^ cells/well to achieve their differentiation. Six days-old MΦ were then exposed for 24 h to various concentration of DEPe during M1 or M2 polarization triggered as described above. Cells were then incubated with 100 μl of MTT solution (0.5 mg/ml) for 2 h at 37°C in a 5% CO_2_ atmosphere. Medium was thereafter discarded and replaced by 100 μl of DMSO. Blue formazan formed products were further quantified by their absorbance at 540 nm using SPECTROstar Nano (BMG Labtech, Ortenberg, Germany).

### Transfection of si RNAs

SMARTpool of individual siRNAs directed against human Nrf2 (si Nrf2) (reference J-003755–12), AhR (si AhR) (reference J-004990–05) and an untargeted sequence (si Ct), used as control, were from Dharmacon (Lafayette, CO, USA). Human MΦ (1 × 10^5^cells/cm^2^) were transfected using Lipofectamine RNA^imax^ (Invitrogen) with 100 nM si Nrf2 for 16 h and 50 nM si AhR for 40 h. MΦ were then polarized into M1 and M2 types and exposed to DEPe for additional 8 h or 24 h. Silencing efficiency of targeted sequences was analyzed by RT-qPCR and Western blot analysis.

### Immunolabelling by flow cytometry

Phenotypic analysis of MΦ was performed using flow cytometric direct immunofluorescence. Cells rinsed in phosphate-buffered saline (PBS) were recovered from culture plates by gently scrapping. They were next incubated for 1 h in PBS with 5% human AB serum at 4°C to avoid nonspecific mAb binding. Several mouse mAbs were then used for immunolabelling: FITC-conjugated mAbs against CD64 (Becton Dickinson Biosciences, Le Pont de Claix, France) and, PE-conjugated mAbs against CD200R and against CD71 provided from eBiosciences SAS (Paris, France). Isotypic control labeling was performed in parallel. Thereafter, cells were analysed with a FC500 flow cytometer (Beckman Coulter, Villepinte, France) using CXP Analysis software (Beckman Coulter). Values were expressed as the ratio of the mean fluorescence intensity (MFI) of the marker of interest over the MFI of the isotype control.

### Endocytosis assay

Briefly, MΦ exposed to DEPe or B(a)P during M1 or M2 polarisation were incubated with 1 mg/ml FITC-dextran wt 40 000 for 60 min at 37°C in a 5% CO_2_ atmosphere. Cellular uptake of FITC-dextran was then monitored by flow cytometry. A negative control was performed in parallel by incubating cells with FITC-dextran at 4°C instead of 37°C. Uptake of FITC-dextran was expressed as ΔMFI, calculated by subtracting MFI measured for uptake at 4°C from that measured for uptake at 37°C, and as % of positive cells, i.e, % positive cells (uptake at 37°C)–% positive cells (uptake at 4°C).

### Quantification of cytokine and chemokine levels

Levels of TNFα, IL-8, IL-10, CXCL10, CCL17, CCL18, CCL22, IL-12p40, IL-12p70, IL-23, and IL-6 secreted in culture medium were quantified by ELISA using specific Duoset ELISA development system kits (R&D Systems).

### RNA isolation and reverse transcription-real time quantitative PCR analysis

Total RNA were isolated from primary MΦ using the TRIzol method (InVitrogen) and were then subjected to reverse transcription-quantitative polymerase chain reaction (RT-qPCR) analysis as previously described [29]. Gene-specific primers, presented in the Table 1, are intron-spanning and purchased from Sigma or as Quantitect (QT) primer assay from Qiagen. Amplification curves of the PCR products were analyzed with the ABI Prism SDS software using the comparative cycle threshold method. Relative quantification of the steady-state target mRNA levels was calculated after normalization of the total amount of cDNA tested to an 18S RNA endogenous reference.

**Table 1:** Primer sequence list.

gene	Name	Forward primer	Reverse primer
18S	ARN 18S	5'-CGCCGCTAGAGGTGAAATTC-3'	5'-TTGGCAAATGCTTTCGCTC-3'
AhR	Aryl hydrocarbon receptor	5'-CTTCCAAGCGGCATAGAGAC-3'	5'-AGTTATCCTGGCCTCGGTTT-3'
BIRC3	Baculoviral IAP Repeat Containing 3	Qiagen PPH0032613–200	
CCL17	CC chemokine type 17	5'-AGCCATTCCCCTTAGAAAGC-3'	5'-CTGCCCTGCACAGTTACAAA-3'
CCL18	CC chemokine type 18	5'-TACCTCCTGGGCAGATTCCAC-3'	5'-CCCACTTCTTATTGGGGTCA-3'
CCL22	CC chemokine type 22	5'-ATTACGTCCGTTACCGTCTG-3'	5'-TAGGCTCTTCATTGGCTCAG-3'
CCL5	CC chemokine type 5	5'-CGCTGTCATCCTCATTGCTA-3'	5'-GCACTTGCCACTGGTGTAGA-3'
CCR7	CC chemokine receptor type 7	5'-GTGGTGGCTCTCCTTGTCAT-3'	5'-TGTGGTGTTGTCTCCGATGT-3'
CD36	Cluster of differentiation 36	5'-AGATGCAGCCTCATTTCCAC-3'	5'-GCCTTGGATGGAAGAACAAA-3'
cox2	Cyclo-oxygénase 2	5'-GAATGGGGTGATGAGCAGTT-3'	5'-GCCACTCAAGTGTTGCACAT-3'
CXCL10	CXC chemokine type 10	5'-CCACGTGTTGAGATCATTGGC-3'	5'-TTCTTGATGGCCTTCGATTC-3'
CXCL11	CXC chemokine type 11	5'-CCTGGGGTAAAAGCAGTGAA-3'	5'-TGGGATTTAGGCATCGTTGT-3'
CYP1B1	Cytochrome P450 1B1	5'TGATGGACGCCTTTATCCTC-3'	5'-CCACGACCTGATCCAATTCT-3'
FABP4	Fatty acid binding protein 4	5'-CCTTTAAAAATACTGAGATTT-3'	5'-GGACACCCCCATCTAAGGTT-3'
GCLm	Glutamate-cysteine ligase regulatory subunit	5'-GCGAGGAGCTTCATGATTGT-3'	5'-CTGGAAACTCCCTGACCAAA-3'
Hmox-1	Heme oxygenase 1	5'-ACTTTCAGAAGGGCCAGGT-3'	5'-TTGTTGCGCTCAATCTCCT-3'
ICAM1	InterCellular Adhesion Molecule 1	Qiagen PPH0046OF-200	
IDO1	Indoleamine-pyrrole 2,3-dioxygenase	5'-GCGCTGTTGGAAATAGCTTC-3'	5'-CAGGACGTCAAAGCACTGAA-3'
IL10	Interleukin-10	5'-CCTGGAGGAGGTGATGCCCCA-3'	5'-CCTGCTCCACGGCCTTGCTC-3'
IL-12p35	Interleukin-12 p35	5'-GATGGCCCTGTGCCTTAGTA-3'	5'-TCAAGGGAGGATTTTTGTGG-3'
IL-12p40	Interleukin-12 p40	5'-CTCGGCAGGTGGAGGTCAGC-3'	5'-TTGCGGCAGATGACCGTGGC-3'
IL-6	Interleukin-6	5'-AGGCACTGGCAGAAAACAAC-3'	5'-TTTTCACCAGGCAAGTCTCC-3'
IL-8	Interleukin-8	5'-AAGAAACCACCGGAAGGAAC-3'	5'-AAATTTGGGGTGGAAAGGTT-3'
LIPA	Lipase A	5'-GGATGAATTCTGGGCTTTCA-3'	5'-TAGCCAGCTCAGGGATCTGT-3'
MRC1	Mannose receptor C type 1	5'-GGCGGTGACCTCACAAGTAT-3'	5'-ACGAAGCCATTTGGTAAACG-3'
NOS3	Nitric oxide synthase 3	Qiagen PPH01298F-200	
NQO1	NAD(P)H dehydrogenase [quinone] 1	5'-GCCGCAGACCTTGTGATATT-3'	5'-TTTCAGAATGGCAGGGACTC-3'
Nrf2	Nuclear factor (erythroid-derived 2)-like 2	5'-AAACCAGTGGATCTGCCAAC-3'	5'-AGCATCTGATTTGGGAATGTG-3'
PPARγ	Peroxisome proliferator-activated receptor gamma	5'-TTCAGAAATGCCTTGCAGTG-3'	5'-CCAACAGCTTCTCCTTCTCG-3'
PTGS1	Cyclooxygenase-1	Qiagen PPH01306F-200	
SLC7A5	Solute carrier family 7 member 5	5'-AATGCATTGGCCTCTGTACC-3'	5'-ACAGGACATGAGCGTGACAG-3'
SR-A1	Scavenger receptor A1	5'-CCTCGTGTTTGCAGTTCTCA-3'	5'-CCATGTTGCTCATGTGTTCC-3'
SR-B1	Scavenger receptor B1	5'-GTGTGGGTGAGATCATGTGG-3'	5'-GTTCCACTTGTCCACGAGGT-3'
TGFβ	Transforming growth factor beta	5'-TGCGCTTGAGATCTTCAAA-3'	5'-GGGCTAGTCGCACAGAACT-3'
TNC	Tenascin C	Qiagen PPH02442A-200	
TNFα	Tumor necrosis factor alpha	5'-AACCTCCTCTCTGCCATC-3'	5'-ATGTTCGTCCTCCTCACA-3'

### Western blot analysis

Cells were harvested and lysed on ice with lysis buffer as previously described [30]. Then, cell lysates were sonicated on ice and protein concentration was quantified using the Bradford's method. Samples were analyzed by 10% SDS-PAGE, and then electroblotted overnight onto nitrocellulose membranes (Bio-Rad). After blocking, membranes were hybridized with primary Abs overnight at 4°C; these primary Abs were directed against AhR (Biomol Research Labs, Plymouth, PA, USA), Nrf2, HSC70 and p38 total (Santa Cruz Biotechnology, USA), knowing that the anti-Nrf2 antibody (H-300) recognizes two bands of Nrf2 around a size of 98–118 KDa[31]. After washing, blots were incubated with appropriate HRP-conjugated secondary Abs. Immunolabelled proteins were finally visualized by autoradiography using chemiluminescence.

### Cell migration assay

Chemotaxis assays were carried out with 12-wells plates exhibiting a 3 μm pore size membrane (Corning, Amsterdam, NDL). Briefly, 3.5 10^5^ M2 MΦ were exposed for 24 h to DEPe during the polarization step as described above to obtain M2 MΦ conditioned medium; 2 × 10^5^ of T-lymphocyte H9 cells, placed in the upper chamber, were allowed to migrate towards MΦ-conditioned media for 4 h at 37°C in a 5% CO_2_ atmosphere. Cells which migrated across the membrane were harvested and subsequently counted by Malassez counting slide. Data were expressed as the number of migrated cells.

### Statistical analysis

The number of subjects and experiments used in each group is stated in the respective figures. Data are expressed as mean ± SEM. Significant differences were evaluated using Student’s t-test or ANOVA followed by the Newman-Keuls multiple comparison test.

## Results

### DEPe effects on polarization marker expression in human M1 and M2 MΦ

**Fig 1:**
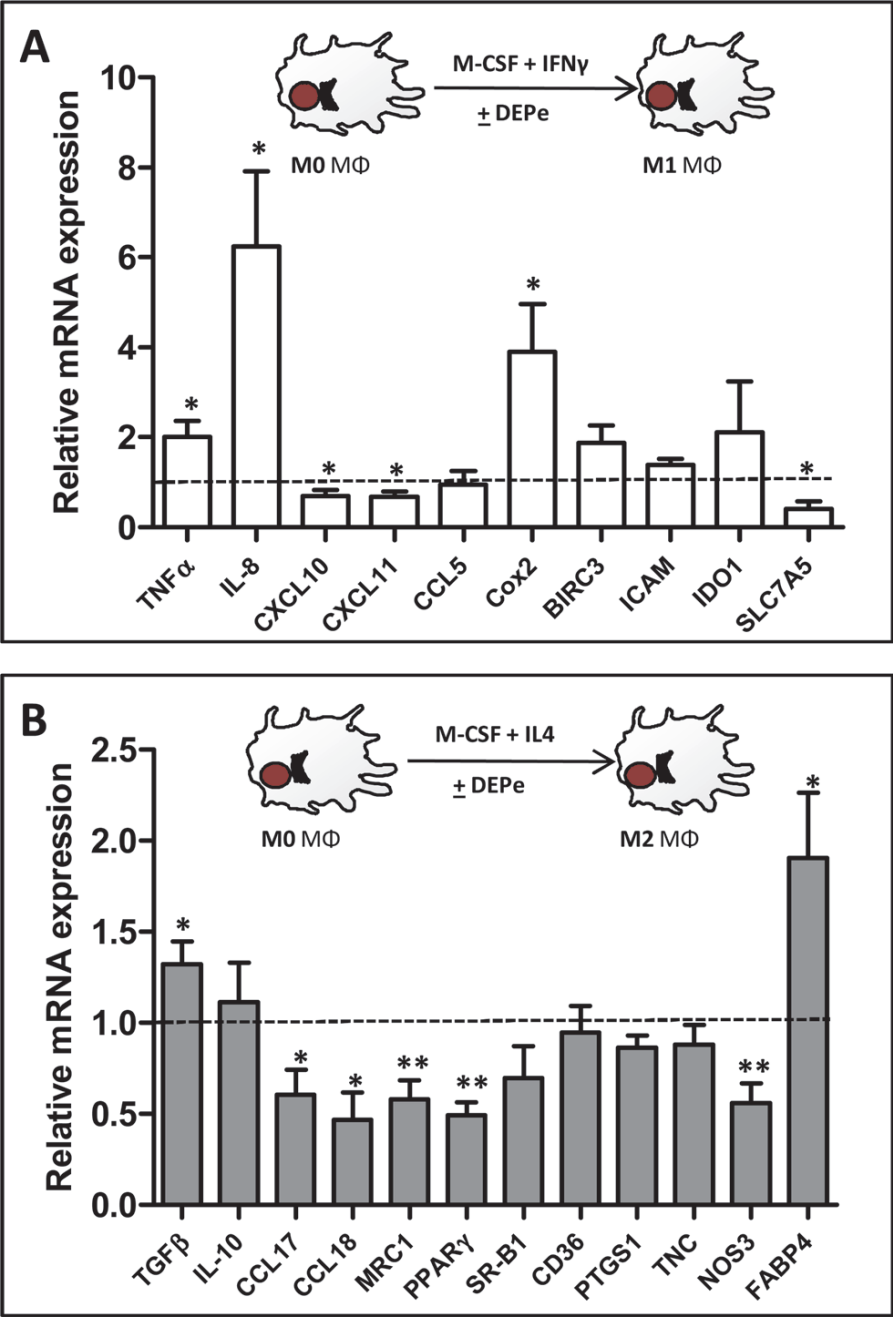
Effects of DEPe on polarization marker mRNA expression during human MΦ polarization. Six-day cultured M-CSF MΦ were activated with IFNγ or with IL-4 to obtain M1 and M2 MΦ, respectively, in the presence of 10 μg/ml DEPe or DMSO during 24 h. Cells were harvested and after total RNA isolation, mRNA levels were determined by RT-qPCR assays. Data are expressed relatively to mRNA levels found in control DMSO-exposed M1 **(A**) or M2 (**B**) MΦ, arbitrarily set at the value of 1 and are the means ± SEM of at least 5 independent experiments. *p<0.05, **p<0.01.

DEPe toxicity towards MΦ was first evaluated using the viability MTT assay; DEPe concentrations resulting in a loss of 50% of viability were found to be similar in M1 (IC_50_ = 59.5 ± 14.4 μg/ml) and in M2 MΦ (IC_50_ = 51.5 ± 12.0 μg/ml), allowing to retain, for further experiments, the non-toxic concentration of 10 μg/ml of DEPe, as attested by the pictures of DEPe-treated macrophages (S1A Fig.). Moreover, a 24-h exposure to this DEPe concentration maximally induced mRNA expression of a reference DEP-target gene, the CYP1B1 (S2 Fig.) [32] [33]. DEPe effects towards a selection of various M1 and M2 macrophagic markers were next analysed by RT-qPCR in MΦ placed in M1 or M2 polarizing conditions. Polarization markers were chosen from our previous results[34] and from the study of Martinez et al. [27] and their validity and relevance in the present study, i.e., their preferential expression in M1 or M2 MΦ, was fully confirmed by RT-qPCR (S3 Fig.). Among the 10 classically activated M1 MΦ markers analysed by RT-qPCR, we found an induction of TNFα, cyclooxygenase-2 (cox-2) and IL-8 and a significant down-regulation of the chemokines CXCL10 and CXCL11 and of the aminoacid transporter SLC7A5 in DEPe-treated M1 MΦ when compared to their untreated counterpart (Fig. 1A); by contrast, mRNA expression of the M1 markers CCL5, BIRC3, ICAM and indoleamine-pyrrole 2,3-dioxygenase (IDO1) remained unchanged in response to DEPe (Fig. 1A). For the 12 alternative activated M2 MΦ markers analysed by RT-qPCR, we found a significant up-regulation of TGF-β and fatty acid binding protein 4 (FABP4), and a down-regulation of CCL17, CCL18, mannose receptor (MRC1), peroxisome proliferator-activated receptor γ (PPARγ) and nitric oxide synthase 3 (NOS3) in DEPe-treated M2 MΦ when compared to their untreated counterparts (Fig. 1B); by contrast, mRNA levels of other typical M2 markers such as IL-10, scavenger receptor B1 (SR-B1), CD36, cyclooxygenase-1 (PTGS1) and tenascin C (TNC) were not modified in M2 MΦ exposed to DEPe (Fig. 1B). Altogether, these data demonstrated that DEPe exposure impaired the acquisition of several markers of classically (M1) and alternative (M2) MΦ polarization, without impairment of MΦ differentiation, as attested by the fact that DEPe failed to abolish the up-regulation of CD71 (S1B Fig.), a marker well-known to be induced during differentiation of monocytes into MΦ. Such DEPe-mediated regulations of M1 and M2 markers were however likely not sufficient in intensity and in the number of regulated genes to overall switch the polarization program of MΦ, i.e., the global profile of M1 and M2 marker mRNA expression was not changed in a major way by DEPe in polarizing MΦ (S3 Fig.). In particular, M1 markers down-regulated by DEPe such as CXCL10, CXCL11 and SLC7A5 remain much more expressed in DEPe-treated M1 MΦ than in M2 MΦ; in the same way, M2 markers down-regulated by DEPe such as CCL17, CCL18, MRC1, PPARγ and NOS3 remain much more expressed in DEPe-treated M2 MΦ than in M1 MΦ (S3 Fig.).

**Fig 2:**
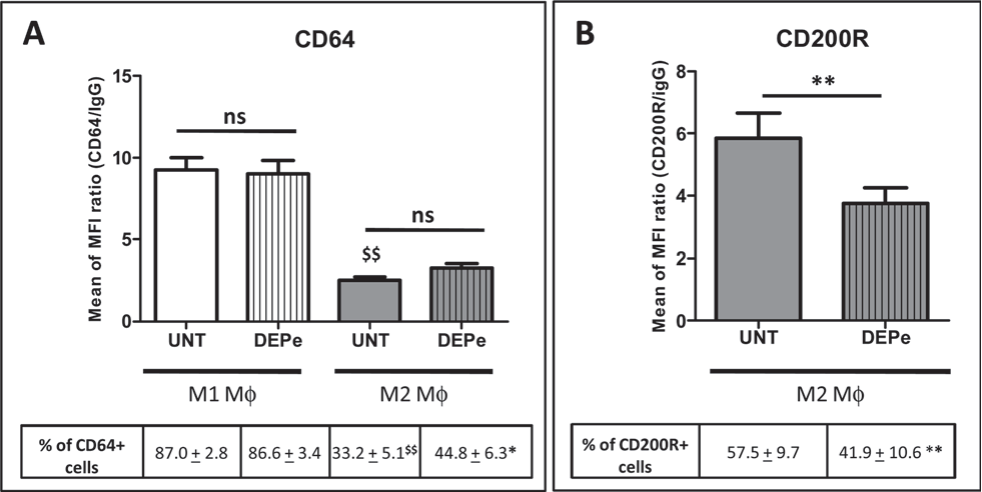
Effects of DEPe on cell surface antigen expression during human MΦ polarization. Six-day cultured M-CSF MΦ were activated with IFNγ or with IL-4 to obtain M1 and M2 MΦ, respectively, in the presence of 10 μg/ml DEPe or DMSO (UNT) during 24 h. Cells were then stained with conjugated mAbs directed against the surface markers CD64 (A) and CD200R (B) and then analyzed by flow cytometry. Histograms represent the means of fluorescence intensity (MFI) ratio ± SEM of 9 independent experiments. *p<0.05 and **p<0.01 when compared with its control counterpart, ^$$^p<0.01 when compared to UNT M1 MΦ; ns: not significant.

Surface expression of the typical M1 marker FCγ receptor 1 (CD64) [35] was next found to be not altered in DEPe-treated M1 MΦ when compared to untreated counterparts, for both MFI ratio and percentage of CD64-positive cells (Fig. 2A). DEPe also failed to change the low expression of this M1 marker in M2 MΦ (Fig. 2A) even if a significant difference in percentage of cells CD64+ was found between untreated and DEP-treated M2 MΦ (Fig. 2A). By contrast, both MFI expression of the inhibitory receptor CD200R, a M2 marker [35] and the percentage of CD200R positive cells were significantly decreased by DEPe in M2 MΦ when compared to their untreated counterparts (Fig. 2B). Expression of CD200R in M1 MΦ was very low, as previously described[34], and was not impaired by DEPe (data not shown).

### DEPe effects on human MΦ functions

We further determined whether the effect of DEPe on MΦ polarization has some functional consequences. For this purpose, the endocytic capacity of M1 and M2 MΦ exposed to DEPe during the polarization step was first measured. The endocytosis of the fluorescent FITC-dextran in DEPe-treated M1 or M2 MΦ was not modified when compared to that found in untreated counterparts, whereas, by contrast, B(a)P-treated M1 and M2 MΦ displayed reduced endocytosis (S4 Fig.), as previously described [36].

**Fig 3:**
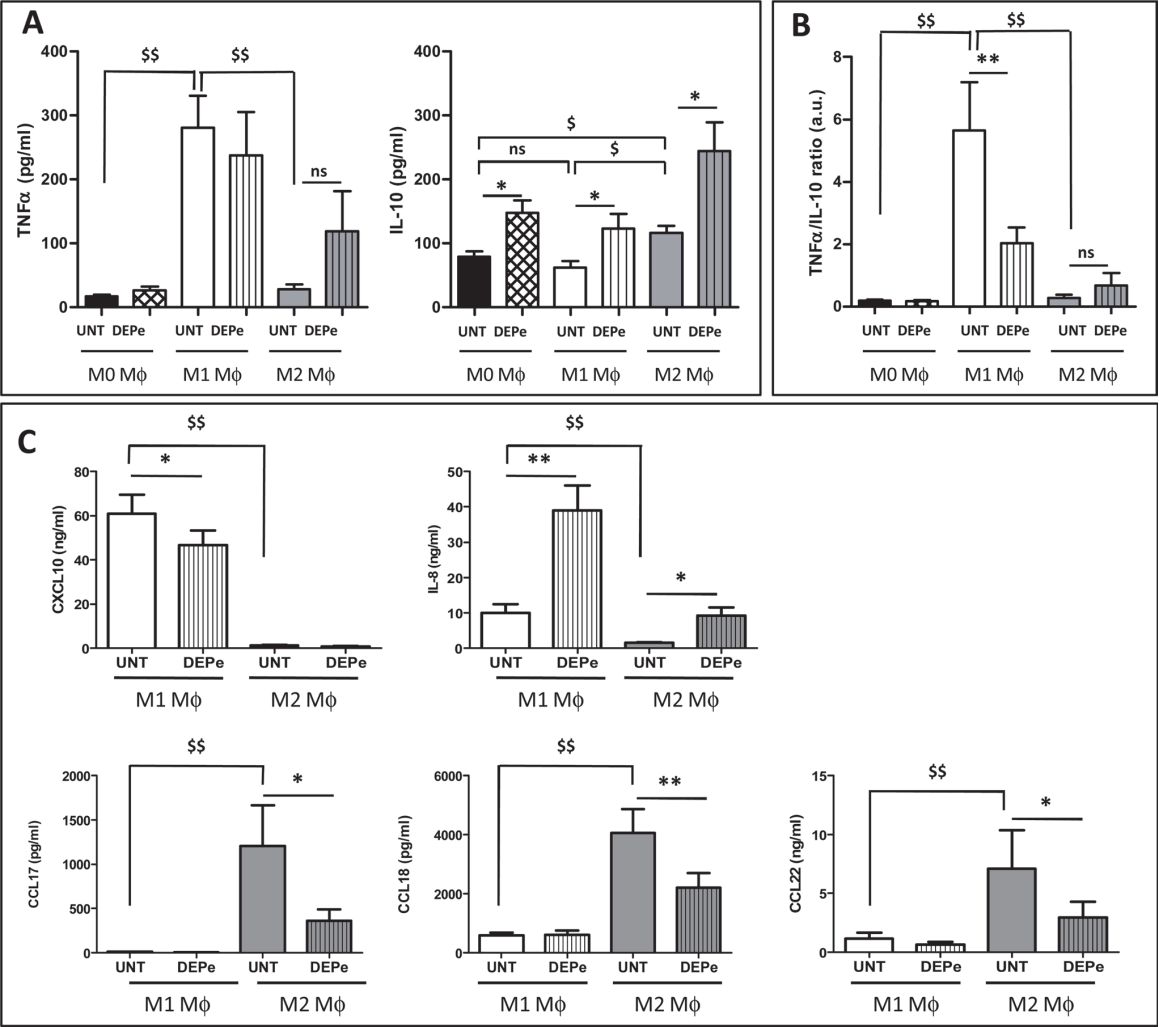
Effects of DEPe on cytokine and chemokine secretion during human MΦ polarization. Six-day cultured M-CSF MΦ were kept unactivated or were activated with IFNγ or with IL-4 to obtain M0, M1 and M2 MΦ, respectively, in the presence of 10 μg/ml DEPe or DMSO (UNT) during 24 h. Cytokine (A, B) and chemokine (C) levels in culture medium were determined by ELISA. (A and C) Data expressed in pg/ml (TNFα, IL-10, CCL17 and CCL18) or ng/ml (IL-8, CXCL10 and CCL22) are the means ± SEM of 8 independent experiments. (B) Data expressed in ratio of TNFα/IL-10 secretion levels (a.u: arbitrary unit) are the means ± SEM of 7 independent experiments. *p<0.05 and **p<0.01 when compared with its control counterpart, ^$$^p<0.01 when compared to M1 UNT MΦ, ns: not significant.

We next measured DEPe effect on cytokine and chemokine secretion capacity by M1 and M2 MΦ. As expected, the levels of the cytokine TNFα were significantly higher in untreated M1 MΦ than in M0 and M2 counterparts (Fig. 3A) and those of the chemokines IL-8 and CXCL10 were significantly higher in untreated M1 MΦ than in M2 counterparts (Fig. 3C). The levels of the cytokine IL-10 was also higher in M2 MΦ than in M0 and M1 counterparts (Fig. 3A) and those of the chemokines CCL17, CCL18 and CCL22 were found significantly higher in untreated M1 MΦ than in M2 counterparts (Fig. 3C); such data, associated to those of mRNA expression (S3 Fig.), fully validated our model of human MΦ polarization. DEPe exposure led to a significant increase of IL-10 secretion in supernatants from M0, M1 and M2 MΦ cultures, whereas TNFα secretion was not significantly modified by DEPe exposure neither in M0, M1 nor in M2 MΦ (Fig. 3A). The ratio of secretion of the pro-inflammatory cytokine TNFα to that of the anti-inflammatory cytokine IL-10 was consequently reduced in M1 MΦ, suggesting an anti-inflammatory impact of DEPe during M1 MΦ polarization (Fig. 3B). Exposure to DEPe was next found to decrease secretion levels of the typical M2 chemokines CCL17, CCL18, CCL22 in M2 MΦ, and of the M1 chemokine CXCL10 in M1 MΦ (Fig. 3C); by contrast, DEPe increased secretion of the M1 marker IL-8 in M1 MΦ and also in M2 MΦ (Fig. 3C).

**Fig 4:**
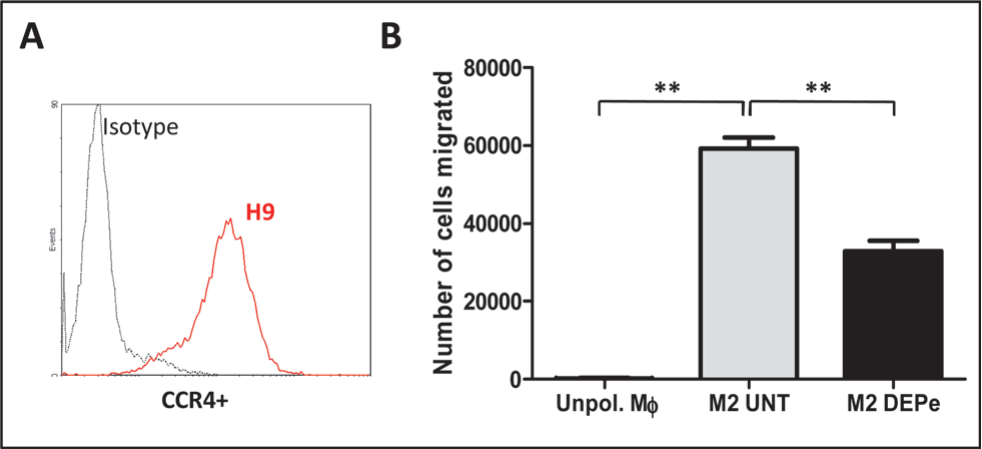
Impact of the down-secretion of CC chemokines by DEPe on the chemotaxis of CCR4+ cells. (A) Graph from flow cytometry showing the CCR4 membrane expression of H9 cells. (B) The number of H9 cells which migrated in the presence of conditioned media of unpolarized MΦ (Unpol.MΦ) or of MΦ exposed to 10 μg/ml DEPe or to DMSO (UNT) during M2 polarization was evaluated by transwell migration assay. Data, representing 3 independent experiments, are expressed in number of cells migrated and are evaluated by cell count. **p<0.01.

As a decrease of CCL17 and CCL22 chemokine secretion was found in culture medium supernatant of M2 MΦ exposed to DEPe, we then wondered if such effect has an impact on chemotaxis of cells expressing CCR4, the surface receptor for CCL17 and CCL22. CCR4-positive lymphoblastic H9 cells (Fig. 4A) strongly migrated in response to conditioned medium from control M2 MΦ, in comparison to cells exposed to conditioned medium of unpolarized MΦ (Fig. 4B); such migration was significantly decreased when conditioned medium of DEPe-exposed M2 MΦ was used (Fig. 4B) demonstrating that DEPe exposure reduced the chemoattactant potential of M2 MΦ.

**Fig 5:**
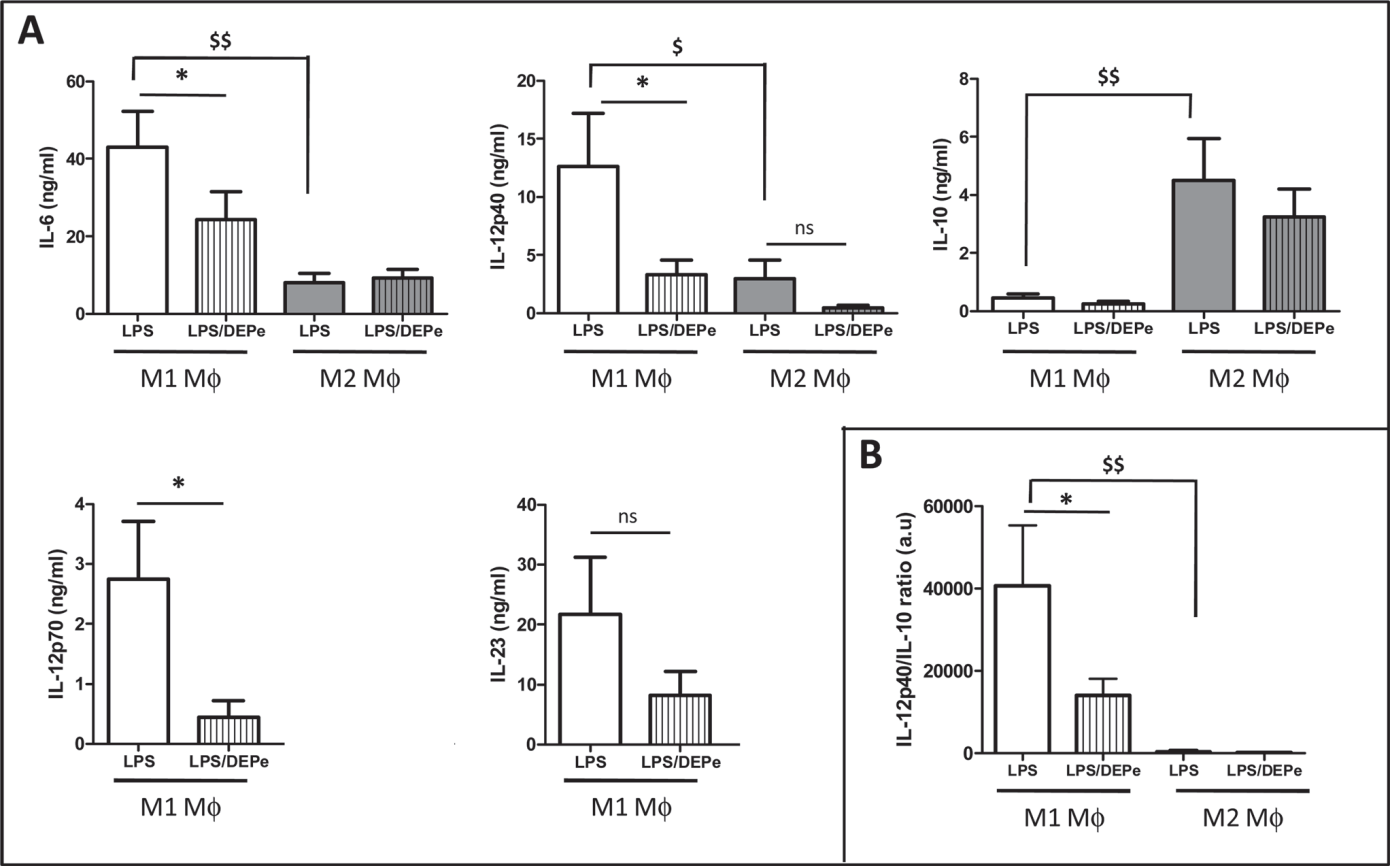
Effects of LPS on cytokine secretion in DEPe-exposed MΦ. Six-day cultured M-CSF MΦ were activated with IFNγ or with IL-4 to obtain M1 and M2 MΦ, respectively, in the presence of 10 μg/ml DEPe or DMSO during 24 h. LPS (10 ng/ml) was next added to DEPe-M1 or DEPe-M2 MΦ for additional 24 h. Cytokine levels in culture medium were determined by ELISA. Data expressed in ng/ml (A) or (B) in ratio of IL-12p40 and IL-10 secretion levels (a.u: arbitrary unit) are the means ± SEM of 6 independent experiments. *p<0.05 when compared with its LPS counterpart, ^$^p<0.05 and ^$$^p<0.01 when compared to M1 LPS MΦ. ns: not significant.

To determine whether DEPe may affect the response of M1 or M2 MΦ to an infectious/inflammatory stimulus, we next exposed untreated and DEPe-treated MΦ to LPS for a further 24-h period. As expected, LPS-exposed M1 MΦ secreted more IL-6 and IL-12p40, the common subunit of IL-12p70 and IL-23, than LPS-exposed M2 MΦ (Fig. 5A). By contrast, IL-10 secretion level was significantly higher in M2 than M1 MΦ in the presence of LPS (Fig. 5A). The concentrations of IL-23 and IL-12p70 in culture media were too low to be detected in M2 MΦ (data not shown). Secretion levels of the cytokines IL-12p40, IL-12p70, IL-23 and IL-6 were all decreased in response to LPS in DEPe-exposed M1 MΦ, whereas those of IL-6 and IL-12p40 remained unaffected by DEPe in M2 MΦ (Fig. 5A). MΦ response to LPS with respect to the secretion of IL-10 was not modified by DEPe exposure, neither in M1 nor M2 MΦ (Fig. 5A). The ratio IL-12p40/IL-10 secretion was consequently reduced by DEPe in M1 subtype (Fig. 5B), likely demonstrating a lower capacity of DEPe-exposed MΦ to respond to LPS.

Finally, we wondered if exposure to DEPe could affect the expression of specific MΦ markers in already polarized MΦ. For this purpose, polarized M1 and M2 MΦ were treated by 10 μg/ml DEPe (S5A Fig.) and surface expression of CD64 and CD200R was first analysed. Similarly to polarizing M1 MΦ (Fig. 2), already polarized M1 MΦ did not exhibit change in CD64 expression in response to DEPe (S5B Fig.); by contrast, CD200R expression was reduced by DEPe in already polarized M2 MΦ (S5B Fig.), as previously described in polarizing M2 MΦ (Fig. 2). With respect to secretion of the cytokine TNFα and the chemokines CXCL10, CCL17, CCL18 and IL-8, treatment of polarized M1 or M2 MΦ by DEPe resulted in regulations (S5C Fig.) similar to those observed in polarizing counterparts (Fig. 3).

### DEPe activates AhR and Nrf2 pathways

**Fig 6:**
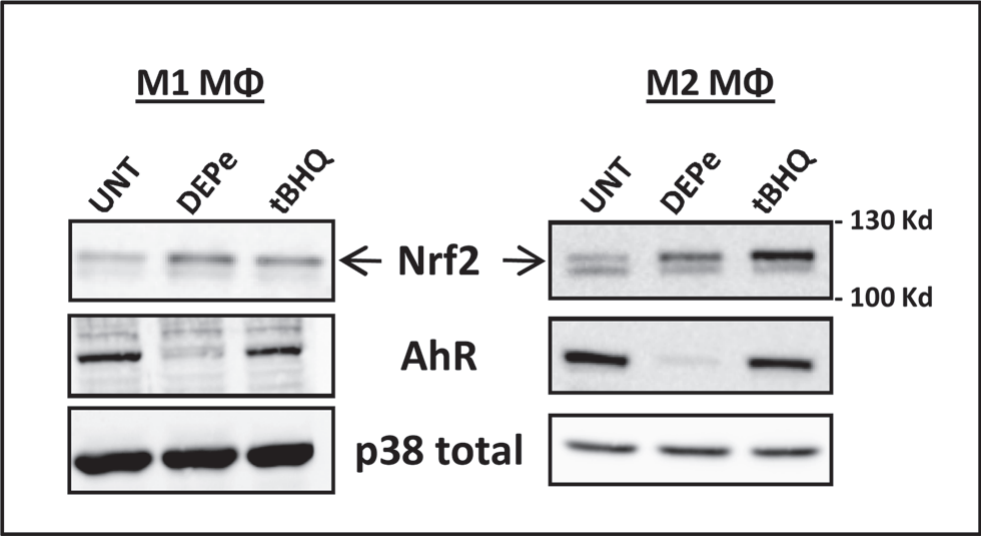
DEPe activates the AhR and Nrf-2 pathways in M1 and M2 MΦ. Six-day cultured M-CSF MΦ were activated with IFNγ or with IL-4 to obtain M1 and M2 MΦ, respectively, in the presence of 10 μg/ml DEPe or 10 μM tBHQ or DMSO (Ct) during 6 h. Western blot analysis of Nrf2 and AhR protein expressions were performed with whole-cell lysates. Equal gel loading and transfer efficiency were checked by blot incubating with Abs against p38 total. Experiments were repeated, 3 times, with similar results.

Signalling pathways putatively involved in DEPe-altered secretion capacity of polarizing MΦ were then analyzed. For this purpose, we studied the putative contribution of AhR and Nrf2 pathways because (i) DEP contain some aryl hydrocarbons well known as AhR ligands, (ii) AhR can interfere with the LPS-TLR4 signalling pathway [37], (iii) DEP leads to nuclear Nrf2 translocation [38] and (iv) exposure to particulate matter results in production of more pro-inflammatory cytokines [21] and CCL17 chemokine [20] in Nrf2-deficient mice than in the wild-type mice. As shown in Fig. 6, a 6-h exposure to DEPe strongly reduced AhR protein expression in both M1 and M2 MΦ, most likely reflecting AhR activation because such activation commonly results in proteosomal degradation of AhR [39]; in parallel, mRNA expression of the CYP1B1, a well-known target of AhR, was increased. DEPe as well as the Nrf2 inducer tBHQ, used here as a reference Nrf2 activator, were also found to increase the expression of the upper band of Nrf2 protein in both M1 and M2 MΦ (Fig. 6), thus likely reflecting Nrf2 activation, because such activation is linked to inhibition of its degradation. Altogether, these results demonstrated that DEPe early activates the AhR and Nrf2 pathways in both M1 and M2 MΦ.

### Involvement of the Nrf2 signalling pathway in DEPe effects towards M1 and M2 macrophagic markers

**Fig 7:**
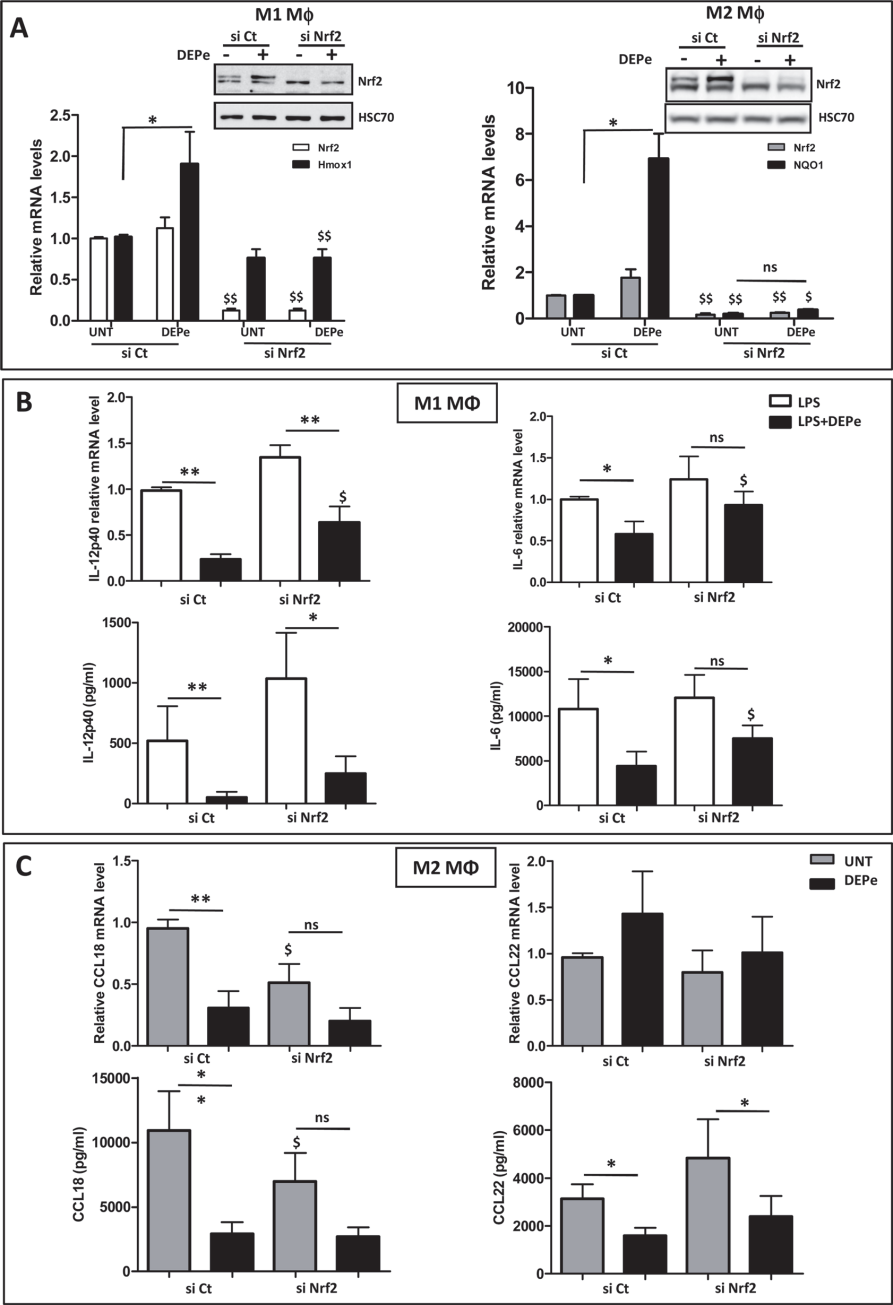
Impact of si Nrf2 on cytokine and chemokine secretions regulated by DEPe in M1 and M2 MΦ. Six-day cultured M-CSF MΦ were transfected with siRNAs targeting Nrf2 (si Nrf2) or with non-targeting siRNAs (si Ct) and cultured for 16 h; they were then activated with IFNγ + LPS or with IL-4 to obtain activated M1 and M2 MΦ, respectively, in the presence of 10 μg/ml DEPe or DMSO (UNT) during 8 h or 24 h. (A) Validation of si Nrf2 efficiency: Western blot analysis of Nrf2 protein expression was performed with whole-cell lysates. Equal gel loading and transfer efficiency were checked by protein hybridization with Abs against HSC70. Experiments were repeated, three times, with similar results. Cells were harvested and after total RNA isolation, mRNA levels of Nrf2 and Hmox-1 in M1 MΦ and Nrf2 and NQO1 in M2 MΦ were determined by RT-qPCR assays. Data are expressed relatively to mRNA levels found in si Ct-transfected cells, arbitrarily set at the value of 1 and are the means ± SEM of at least 4 independent experiments. (B) Cytokine mRNA expression and secretion in culture medium of M1 MΦ were determined respectively by RT-qPCR and by ELISA after 8h. (C) Chemokine mRNA expression and secretion in culture medium of M2 MΦ were determined respectively by RT-qPCR and by ELISA after 24h. Data are the means ± SEM of at least 5 independent experiments. *p<0.05 and **p<0.01 when compared with its untreated counterpart; ^$^p<0.05 and ^$$^p<0.01 when compared to their respective si Ct-transfected counterparts; ns: not significant.

To further determine the role of Nrf2 in DEPe effects towards MΦ, we down-regulated the expression of Nrf2 by RNA interference. The transient transfection of RNAi against Nrf2 (si Nrf2) reduced endogenous and DEP-inducible Nrf2 protein expression in M1 and M2 MΦ in comparison to cells transfected with a non-targeting siRNA (si Ct) used as control (Fig. 7A, insert); the efficiency of si Nrf2 transfection was also confirmed by the down-expression of Nrf2 mRNA levels and of Nrf2 target genes Hmox-1 in M1 MΦ or NQO1 in M2 MΦ (Fig. 7A). DEPe significantly reduced LPS-induced IL-12p40 expression at both mRNA and protein levels in si Ct- and si Nrf2-transfected M1 cells (Fig. 7B). By contrast, DEPe significantly reduced the LPS-induced IL-6 expression at both the mRNA and protein levels only in si Ct-transfected cells, demonstrating that the lowest IL-6 secretion in DEPe-exposed M1 MΦ in response to LPS is Nrf2-dependent. In M2 MΦ, the absence of Nrf-2 reduces significantly CCL18 mRNA and secretion levels in untreated condition, suggesting a potential role of this transcription factor in CCL18 basal expression (Fig. 7C). The decrease of CCL18 mRNA and secretion levels was found significant in DEPe-treated si Ct-transfected cells but not in DEPe-treated si Nrf2-transfected cells, probably because of the reduced basal levels of CCL18 in untreated si Nrf2 transfected cells (Fig. 7C). In order to clarify the role of Nrf2, we compared the repression factors of the CCL18 secretion after DEPe exposure (4.5 ± 0.81 fold in si Ct-transfected cells and 2.9 ± 0.49 fold in si Nrf2-transfected cells) and we found no significant difference between these repression factors, suggesting that DEPe effects on CCL18 secretion is probably Nrf2-independent. The significant decrease of CCL22 secretion observed in si Ct-transfected M2 MΦ was also well found in the si Nrf2-transfected M2 MΦ after DEPe exposure (Fig. 7C), suggesting that DEPe effects on CCL22 secretion are Nrf2-independent; moreover, mRNA levels of CCL22 were not modified by DEPe-treatment (Fig. 7C), indicating that DEPe effects towards CCL22 are likely post-transcriptional.

### Involvement of the AhR signalling pathway in DEPe effects towards M1 and M2 macrophagic markers

**Fig 8:**
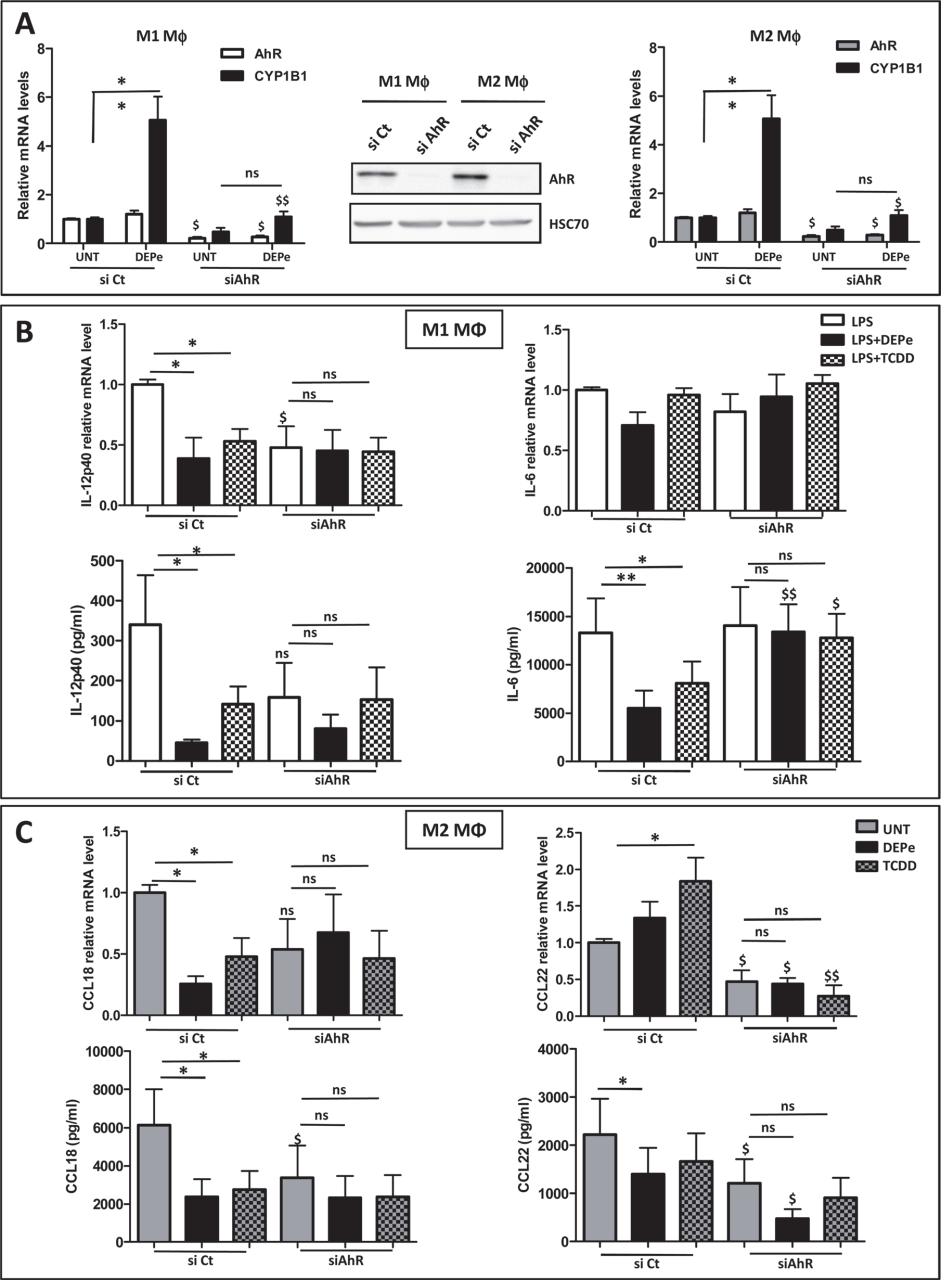
Impact of si AhR on cytokine and chemokine secretions regulated by DEPe in M1 and M2 MΦ. Six-day cultured M-CSF MΦ were transfected with siRNAs targeting AhR (si AhR) or with non-targeting siRNAs (si Ct) and cultured for 40 h; they were activated with IFNγ ± LPS or with IL-4 to obtain LPS activated M1 MΦ and M2 MΦ, respectively, in the presence of 10 μg/ml DEPe, 10 nM TCDD or DMSO (UNT) during 8 h or 24 h. (A) Validation of si AhR efficiency: Western blot analysis of AhR protein expressions were performed with whole-cell lysates. Equal gel loading and transfer efficiency were checked by protein hybridization with Abs against HSC70. Experiments were repeated, three times, with similar results. Cells were harvested and after total RNA isolation, mRNA levels of AhR and CYP1B1 were determined by RT-qPCR assays. Data are expressed relatively to mRNA levels found in si Ct-transfected cells, arbitrarily set at the value of 1 and are the means ± SEM of at least 4 independent experiments. (B) Cytokine mRNA expression and secretion in culture medium of M1 MΦ were determined respectively by RT-qPCR and by ELISA after 8 h. (C) Chemokine mRNA expression and secretion in culture medium of M2 MΦ were determined respectively by RT-qPCR and by ELISA after 24h. Data are the means ± SEM of at least 4 independent experiments. *p<0.05 and **p<0.01 when compared with its untreated counterpart; ^$^p<0.05 and ^$$^p<0.01 when compared to their respective si Ct-transfected counterparts; ns: not significant.

To further determine the role of AhR in DEPe effects towards M1 and M2 markers, we down-regulated the expression of AhR by RNA interference. As shown on the Western blot of the Fig. 8A, the transient transfection of RNAi against AhR (si AhR) reduced endogenous AhR protein expression in comparison to cells transfected with non-targeting siRNA (si Ct) in both M1 and M2 MΦ; the efficiency of si AhR transfection was also confirmed by the down-expression of AhR mRNA levels and by the inhibition of DEPe-mediated induction of CYP1B1 in M1 and M2 MΦ (Fig. 8A). DEPe and TCDD, used here as a reference AhR ligand, reduced significantly LPS-induced IL-12p40 expression at both mRNA and protein levels in si Ct-transfected M1 cells but not in si AhR-transfected M1 cells (Fig. 8B). DEPe and TCDD also reduced significantly LPS-induced IL-6 secretion in si Ct-transfected M1 cells but not in si AhR-transfected M1 cells (Fig. 8B). We also observed that AhR seems to regulate basal secretion of IL-12p40 but not that of IL-6 in M1 MΦ. In M2 MΦ, DEPe and TCDD reduced significantly CCL18 expression at both mRNA and protein levels in si Ct-transfected cells but not in si AhR-transfected counterparts (Fig. 8C). The significant decrease of CCL22 secretion by DEPe exposure in si Ct-transfected cells was not confirmed at mRNA level, suggesting a post-transcriptional effect of DEPe on CCL22 expression as already indicated above. Similarly, TCDD exposure tends to decrease CCL22 secretion but not those of mRNA expression (Fig. 8C). Interestingly, basal secretions of CCL18 and CCL22 chemokines seem to require the presence of AhR because their levels were found to be decreased in untreated si AhR-transfected M2 MΦ (Fig. 8C). Altogether, these data suggest that DEPe effects on IL-12p40 and IL-6 secretion in LPS-stimulated M1 MΦ and on CCL18 secretion in M2 MΦ require AhR.

## Discussion

In the current study, we investigated the effects of DEPe on the acquisition of polarization markers in human M1 and M2 MΦ and the role of AhR and Nrf2 in these processes. Our results showed that 1) M1 and M2 human MΦ are both sensitive to DEPe; 2) DEPe effects seem gene-specific and not dependent on the type of MΦ polarization; 3) DEPe decreases the expression of the CD200R, a typical membrane marker of M2 MΦ; 4) DEPe decreases the capacity of M1 polarizing MΦ to secrete the pro-Th1 chemokine CXCL10 and the LPS-induced IL-6 and IL-12p40 cytokines; 5) the decreased capacity of M2 polarizing MΦ exposed to DEPe to secrete CCL17, CCL18 and CCL22 chemokines is associated to a lower chemotaxis of CCR4+ cells; 6) AhR and Nrf2 pathways are activated in both types of MΦ after DEPe exposure; 7) the DEPe-related decrease of IL-6 secretions is Nrf2- and AhR-dependent; 8) the DEPe-related decreases of IL-12p40 and CCL18 secretion are AhR-dependent but Nrf2-independent.

About fifty percent of the specific polarization genes that we have studied in the present study are regulated by DEPe in M1 and M2 MΦ (with a majority of down-expression), indicating that DEPe exposure during the polarisation step alters the acquisition of some macrophagic markers. We can reasonably exclude an inhibitory effect of DEPe on IFNγ or IL-4 activation pathways, because the expression of some macrophagic markers under control of these factors is increased or unmodified by the pollutant. Likewise, the phosphorylation of STAT-1 by IFN-γ in M1 MΦ and the phosphorylation of STAT-6 by IL-4 in M2 MΦ were not altered in the presence of DEPe in the first four hours of polarization (data not shown). The fact that some DEPe effects observed during the polarization step of MΦ were also confirmed on already polarized MΦ also supports the idea that DEPe effects that we observed are independent of agonist-induced polarization program (S5 Fig.). Therefore, our data reveal that DEPe effects are gene-specific and less linked to a family of gene or to a type of MΦ, and can therefore not reflect an overall alteration of M1 and M2 differentiation processes.

In mammals, the first line of defence includes antigen recognition by pattern recognition receptors such as Toll-like receptors, scavenger receptors (SR) and mannose receptors (MR). DEPe exposure reduced the mRNA expression of MRC1 and, to a lesser extent SR-B1, but not those of CD36 in M2 MΦ. Although the proteins encoded by these genes are involved in endocytosis, this function is not affected by DEPe in contrast to B(a)P, suggesting that their membrane expressions are not altered by DEPe exposure and remain functional.

The functions of MΦ depend largely of their ability to secrete mediators such as cytokines and chemokines. Thus, the decreased secretion of the pro-inflammatory cytokines IL-6 and IL-12p40 by DEPe that we observed in a context of TLR4 activation in M1 MΦ and of TNFα in LPS-stimulated human alveolar MΦ [9] will probably reduce their capacities to activate B and T lymphocytes and NK cells and therefore support the notion that DEP can reduce host defence, especially resistance to infection. In this context, it is noteworthy that diesel-enhanced influenza infection in lung mice was found to be associated with a decrease of IL-12p40 [40]. It was also demonstrated that DEPe suppressed Listeria monocytogenes-induced secretion of IL-12 and TNFα in rat alveolar MΦ and that DEP-enhanced production of IL-10 may also increase the susceptibility of diesel particulate matter-exposed MΦ to bacterial infection [41]. Moreover, by decreasing the pro-Th1 chemokine CXCL10 secretion in M1 MΦ, DEPe can also reduce the chemotaxis of CXCR3-positive cells such as type 1 (Th1) T cells, exacerbating the decrease of T cell activation and may indirectly favour a type-2 response. This CXCL10 decrease is consistent with that found in DEP-treated monocyte-derived dendritic cells [42] and in PBMC of nonatopic subjects exposed to DEP-PAH [23]. Successful clearing of a respiratory bacterial infection depends on an adequate Th1 immune response; therefore, the subject would not control the infection as well if exposed to particulate matter. By contrast, DEPe-induced IL-8 secretion in both types of MΦ strongly suggests that neutrophil recruitment would be efficient in DEP-exposed tissue, in accordance with the lung inflammation observed in DEP-exposed subjects [43–44].

In addition, by decreasing CCL17 and CCL22 secretion capacity of M2 MΦ, DEPe may indirectly reduce chemotaxis of CCR4-positive cells such as type 2 (Th2) T cells and basophils, both involved in the allergic immune response [45–46]. Moreover, the decrease of CCL18 secretion after DEPe exposure may alter the chemoattractive capacity of this chemokine toward several cell types; thus CCL18 which is constitutively expressed at high level in the lung and mainly by alveolar MΦ acts as a chemoattractant for naive CD4+ T lymphocytes and immature monocyte-derived dendritic cells, which may lead to the development of tolerogenic immune response. The chemokines CCL17, CCL18 and CCL22 could also play a crucial role in maintaining lung tolerance by attracting Tregs into lung [47]. Therefore, we can presume that a reduction of such chemokine secretion by DEP exposure would lead to a decrease of lung immune response.

Although the AhR was initially recognized as a receptor of several pollutants such as PAHs, AhR by itself is now recognized to play an important role in the control of the adaptive immune response [14, 48]. Moreover, immunity- and inflammation-related functions were found up-regulated in response to the AhR agonist B(a)P in primary human MΦ [49] and various cytokines and chemokines have been shown to be targeted by AhR [13]. So, B(a)P has been shown to induce IL-8 expression in both human MΦ or in mouse lung [50], but AhR activation is also able to inhibit LPS-induced IL-1β, IL-6 or IL-12 expression in MΦ or dendritic cells [51, 13]. Here, we report that the activation of AhR by DEPe is involved in the lower capacity of human MΦ to secrete some specific cytokines of M1 subtype such as IL-6 and IL-12p40 and the specific CCL18 chemokine specific of the M2 subtype. Importantly, the basal level of AhR and its activation by DEPe was not found to differ in both M1 and M2 MΦ, thus likely discarding a role for AhR in MΦ polarization. IL-6 and IL-12 are both regulated by NF-κB and the mechanisms by which AhR exerts its anti-inflammatory activity could be related to its capacity to interact with RelB, a member of the NF-κB family [52], with STAT-1 [37] or with Sp1 [53] after LPS stimulation as previously described in mouse MΦ. Among the CC chemokines family, AhR has been shown to be involved in the up-regulation of CCL1 after B(a)P [54] or TCDD [55] exposure in human MΦ but also in the down-regulation of CCL5 after an exposure to a AhR ligand *in vivo* [56] and in the human keratinocyte Hacat cell line [57]. However, it is the first time to our knowledge that the CCL18 chemokine was found regulated by DEP and by AhR. Such down-regulation of CCL18 previously described in alveolar MΦ of cigarette smokers [58] therefore suggests that some common components of these two air pollutants (DEP and cigarette smoke), such as PAHs, could be involved in CCL18 down-regulation via AhR activation. Despite AhR knock-down has reversed DEP-induced CCL22 down-secretion, the role of AhR in such regulation is not clear; thus, the mRNA and secretion levels were not well correlated and the referent AhR ligand TCDD failed to decrease the CCL22 secretion, knowing moreover that CCL22 was previously found up-regulated by DEPe in alveolar MΦ from allergic patients [59]. Interestingly, an *in silico* analysis has permitted the identification of some XREs in the promoter regions of the CCL17, CCL18 and CCL22 genes [60]; however the functionality of these XRE sites has not been investigated and we cannot exclude a regulation of the chemokines by AhR independently of XRE. Moreover, the mechanisms leading to a decrease of CCL17 and CCL22 production in the human keratinocyte Hacat cell line would be associated to an overexpression of HO-1 [61].

Nrf2, one of the principal regulators of the cellular line of defense, was well activated after DEPe exposure in human M1 and M2 MΦ; such activation was validated by the overexpression of some stress defense genes as Hmox-1 and NQO1. In parallel to its role in defense against oxidative stress, Nrf2 has been shown to suppress pro-inflammatory signaling pathway [16]. Thus, in respiratory models, DEP caused a significantly enhanced airway responsiveness and eosinophilic inflammation in Nrf2-deficient mice [20] and, in models of chronic obstructive pulmonary disease, Nrf2-deficient mice have increased recruitment of neutrophils and MΦ to the lung [62]. In this study, we demonstrated that the down-expression of Nrf2 permits to maintain the level of LPS-induced IL-6 secretion in M1 MΦ but not of IL-12p40. These results contrast to previous studies showing an increase of both IL-6 and IL-12p40 secretion in a model of ventilator-induced lung injury in Nrf2-deficient mice [63], and demonstrating that DEP-reduced LPS induction of IL-12p40 in human dendritic cells involves Nrf2 [23]. These differences may be related to some variations in regulatory pathways between species or the cellular models. The mechanisms by which Nrf2 controls the expression levels of IL-6 after DEPe exposure could be related to an activation of the nuclear factor NF-κB observed after a disruption of Nrf2 in a murine model of sepsis[19]. The role of ATF3, a TLR- and a ROS-inducible transcription factor, which acts a negative regulator of IL-6 and IL-12p40 in mice [64–65] needs to be accurately studied, because DEPe seems to increase its mRNA expression in our models of MΦ (data not shown). Recently, the anti-inflammatory effect of Nrf2 on IL-6 secretion in LPS-stimulated MΦ was proposed to be connected to AMP-activated protein kinase (AMPK) activation [66]. To our knowledge, the DEP effects on AMPK activation has not been studied, but we previously demonstrated that 1-nitropyrene, a nitro-polycyclic aromatic hydrocarbon present in diesel exhaust, increases AMPK activity [67], thus suggesting that AMPK way could be an another regulatory way of IL-6 by DEP. The role of Nrf2 on the CCL18 chemokine secretion is not easy to determine because of the decrease of the basal level of secretion in Nrf2-deficient cells. Therefore, we propose that IL-4-induced CCL18 could be Nrf2-dependent and that the down-regulation of DEPe on CCL18 but also on CCL22 could be Nrf2-independent. A down-expression of CCL22 in MΦ by the inorganic arsenic which activates Nrf2, has previously been related to a decrease of the transcriptional factor Egr2 expression [68], which is moreover more expressed in human M2 MΦ than M1 MΦ [34]; however, we do not found a similar Egr2 regulation by DEPe in M2 MΦ (data not shown).

The DEPe concentration used (10 μg/ml) in the present study was selected on the basis of the absence of cell toxicity and on previous studies realized on MΦ [69, 9]. This concentration of DEPe seems to be equivalent to an *in vitro* particles deposition of 2 μg/cm^2^ and corresponds to a 24 h exposure to a polluted urban environment [23]. However, the effects of DEPe on the mRNA expression of some activated macrophages (CXCL10, CCL18 and IL-8) start from 1–5 μg/ml (S2 Fig.) suggesting that macrophage polarization could be altered at lower concentration than 10 μg/ml of DEPe. Although the effects of DEPe do not accurately reflect those of the whole particulates, it seems that the alteration of MΦ functions could appear at doses of DEP commonly reached in urban areas or during peak pollution, highlighting the putative relevance of our *in vitro* data to environmental exposure.

In summary, this study demonstrates that *in vitro* exposure of polarizing human MΦ to DEPe significantly reduces the acquisition of several M1 and M2 markers of MΦ activation and the response of M1 MΦ to LPS, as monitored by the reduction of IL-12p40 and IL-6 secretion. It further indicates that AhR and Nrf2 are involved in such effects. Through altering some M1 and M2 marker expression, DEP, which constitute the main atmospheric pollutant in urban areas, may alter the immune response of individuals exposed to these atmospheric pollutants and may therefore increase their susceptibility to respiratory infections.

## Supporting Information

S1 Fig Phenotypic effects of DEPe. Six-day cultured M-CSF MΦ were unpolarized or activated with IFNγ or with IL-4 to obtain M1 and M2 MΦ, respectively, in the presence of 10 μg/ml DEPe during 24 h. (A) Morphological feature of DMSO- or DEPe-exposed MΦ (Phase-contrast microscopy, magnification x200) (B) Cells were then stained with conjugated mAbs directed against the surface markers CD71 and then analyzed by flow cytometry. Data are representative of 2 independent experiments.
(TIF)

S2 Fig Effects of various DEPe concentrations on polarization marker mRNA expression during human MΦ polarization. Six-day cultured M-CSF human primary were activated with IFNγ or with IL-4 to obtain classically activated MΦ (M1) or alternative activated MΦ (M2), respectively, in the presence of indicated doses of DEPe (μg/ml) during 24 h. CYP1B1, IL-8, CXCL10 and CCL18 mRNA level was estimated by RT-qPCR; data were expressed relatively to mRNA levels found in control DMSO-exposed cells, arbitrarily set at the value of 1 unit and are the means ± SEM of at least 3 independent experiments. *p<0.05.
(TIF)

S3 Fig Comparison of DEPe effects on polarization marker mRNA expression during human MΦ polarization. Six-day cultured M-CSF MΦ were activated with IFNγ or with IL-4 to obtain M1 and M2 MΦ respectively, in the presence of 10 μg/ml DEPe or DMSO (UNT) during 24 h. Cells were harvested and after total RNA isolation, mRNA levels were determined by RT-qPCR assays. Quantification of the steady-state target mRNA levels was calculated after normalization of the total amount of cDNA tested to an 18S RNA endogenous reference, using the 2^-Ct^ method. This allowed to get a value of expression for each gene specific of M1 (A) or M2 (B) activation, comparatively to the 18S RNA amount found in RT-qPCR sample, which is presumed to remain constant between the different samples and which was arbitrarily set at 10^7^ units (a.u) (Moreau *et al*., 2011). Data are the means ± SEM of at least 4 independent experiments. *p<0.05, **p<0.01.
(TIF)

S4 Fig Effects of DEPe on endocytosis. Six-day cultured M-CSF MΦ were activated with IFNγ or with IL-4 to obtain M1 and M2 MΦ, respectively, in the presence of 10 μg/ml DEPe, 2 μM B(a)P or DMSO (UNT) during 24 h. MΦ were incubated with FITC-dextran at 4°C (negative control) or 37°C to measure endocytosis. Cellular uptakes of FITC-dextran, determined by flow cytometry, are expressed as ∆MFI (∆MFI = MFI _37°C_—MFI _4°C_) and are the means of 7 independent experiment. * p<0.05 when compared with untreated MΦ. Ns: not significant.
(TIF)

S5 Fig Effects of DEPe on polarization marker expression and cytokine and chemokine secretion on already polarized M1 and M2 human MΦ. (A) Six-day cultured M-CSF MΦ were activated during 24 h with IFNγ or with IL-4 to obtain M1 and M2 MΦ, respectively. MΦ were then exposed to 10 μg/ml DEPe or to DMSO (UNT) for additional 24 h. (B) Supernatants were collected and cells were then stained with conjugated mAbs directed against the surface markers CD64 and CD200R to be analyzed by flow cytometry. Histograms represent the means of fluorescence intensity (MFI) ratio ± SEM of 7 independent experiments; **p<0.01. (C) Cytokine and chemokine levels in culture medium were determined by ELISA. Data expressed in pg/ml are the means ± SEM of 5 independent experiments. *p<0.05, **p<0.01, ns: not significant.
(TIF)
