# Supplementary figures and images for: Exposure to Diesel Exhaust Particle Extracts (DEPe) Impairs Some Polarization Markers and Functions of Human Macrophages through Activation of AhR and Nrf2
Source: PLoS One. 2015 Feb 24;10(2):e0116560. doi: 10.1371/journal.pone.0116560 (PMC4339390; doi:10.1371/journal.pone.0116560)

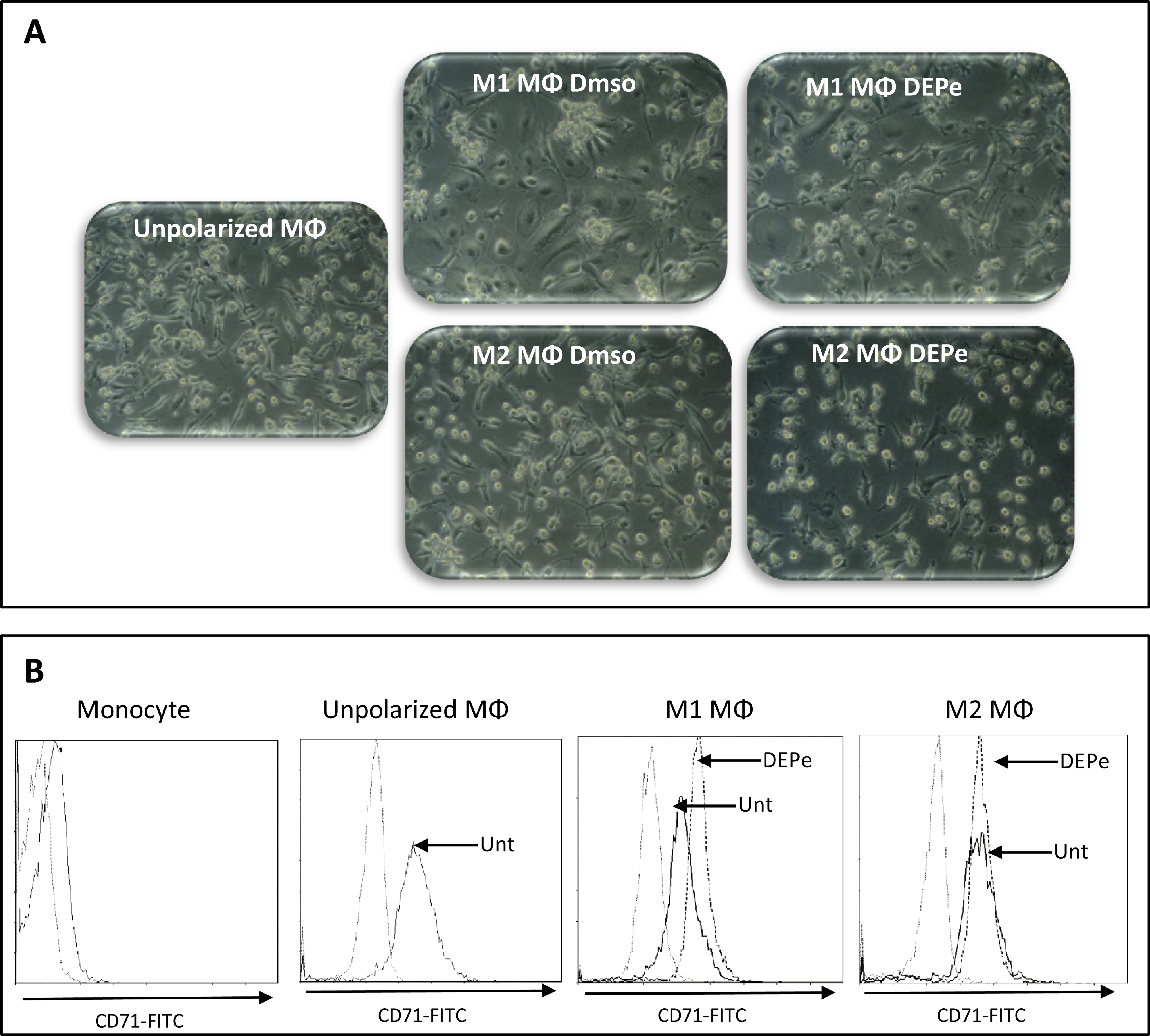

Supplement: S1 Fig — Six-day cultured M-CSF MΦ were unpolarized or activated with IFNγ or with IL-4 to obtain M1 and M2 MΦ, respectively, in the presence of 10 μg/ml DEPe during 24 h. (A) Morphological feature of DMSO- or DEPe-exposed MΦ (Phase-contrast microscopy, magnification x200) (B) Cells were then stained with conjugated mAbs directed against the surface markers CD71 and then analyzed by flow cytometry. Data are representative of 2 independent experiments. (TIF) [file pone.0116560.s001.tif]

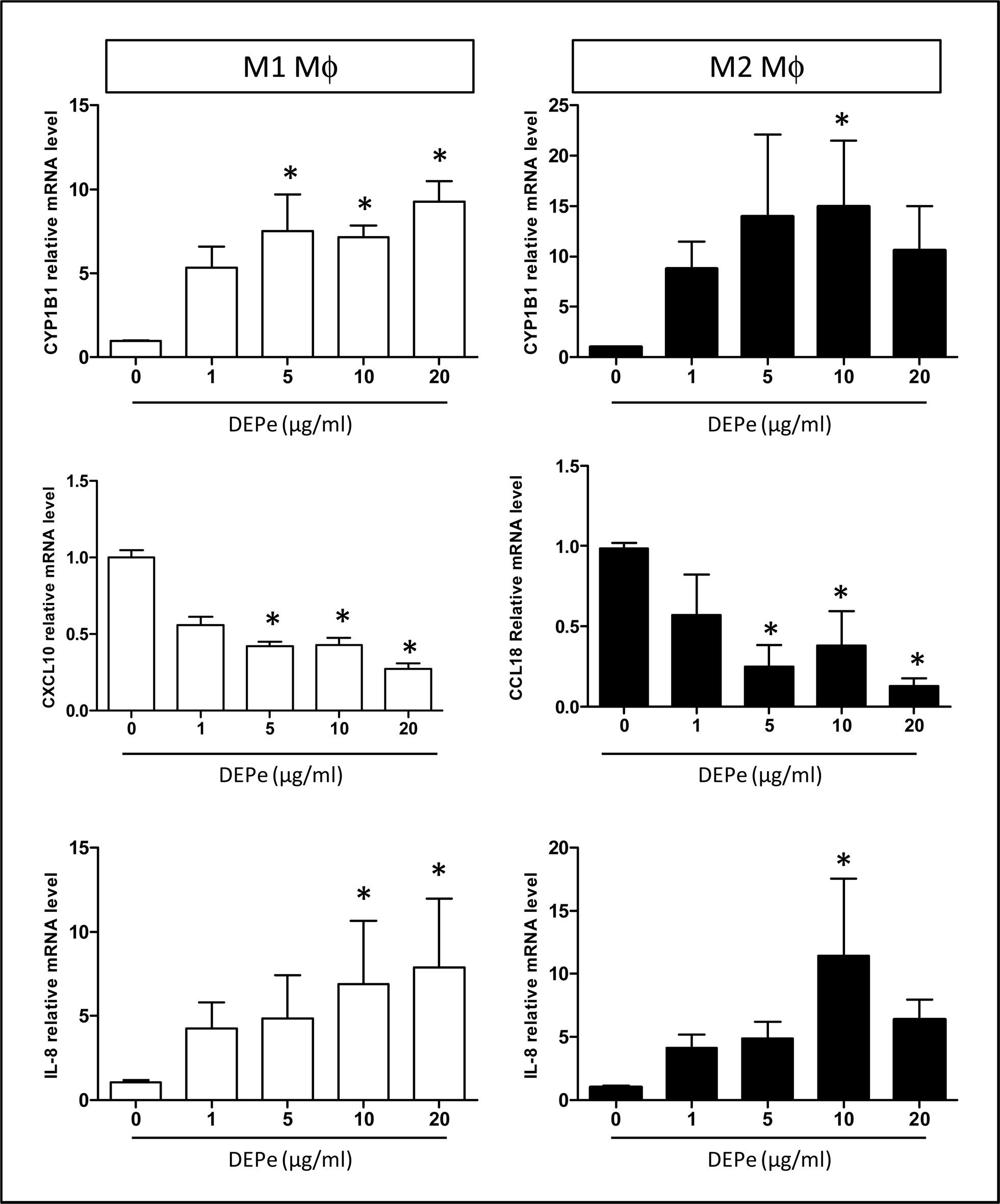

Supplement: S2 Fig — Six-day cultured M-CSF human primary were activated with IFNγ or with IL-4 to obtain classically activated MΦ (M1) or alternative activated MΦ (M2), respectively, in the presence of indicated doses of DEPe (μg/ml) during 24 h. CYP1B1, IL-8, CXCL10 and CCL18 mRNA level was estimated by RT-qPCR; data were expressed relatively to mRNA levels found in control DMSO-exposed cells, arbitrarily set at the value of 1 unit and are the means ± SEM of at least 3 independent experiments. *p<0.05. (TIF) [file pone.0116560.s002.tif]

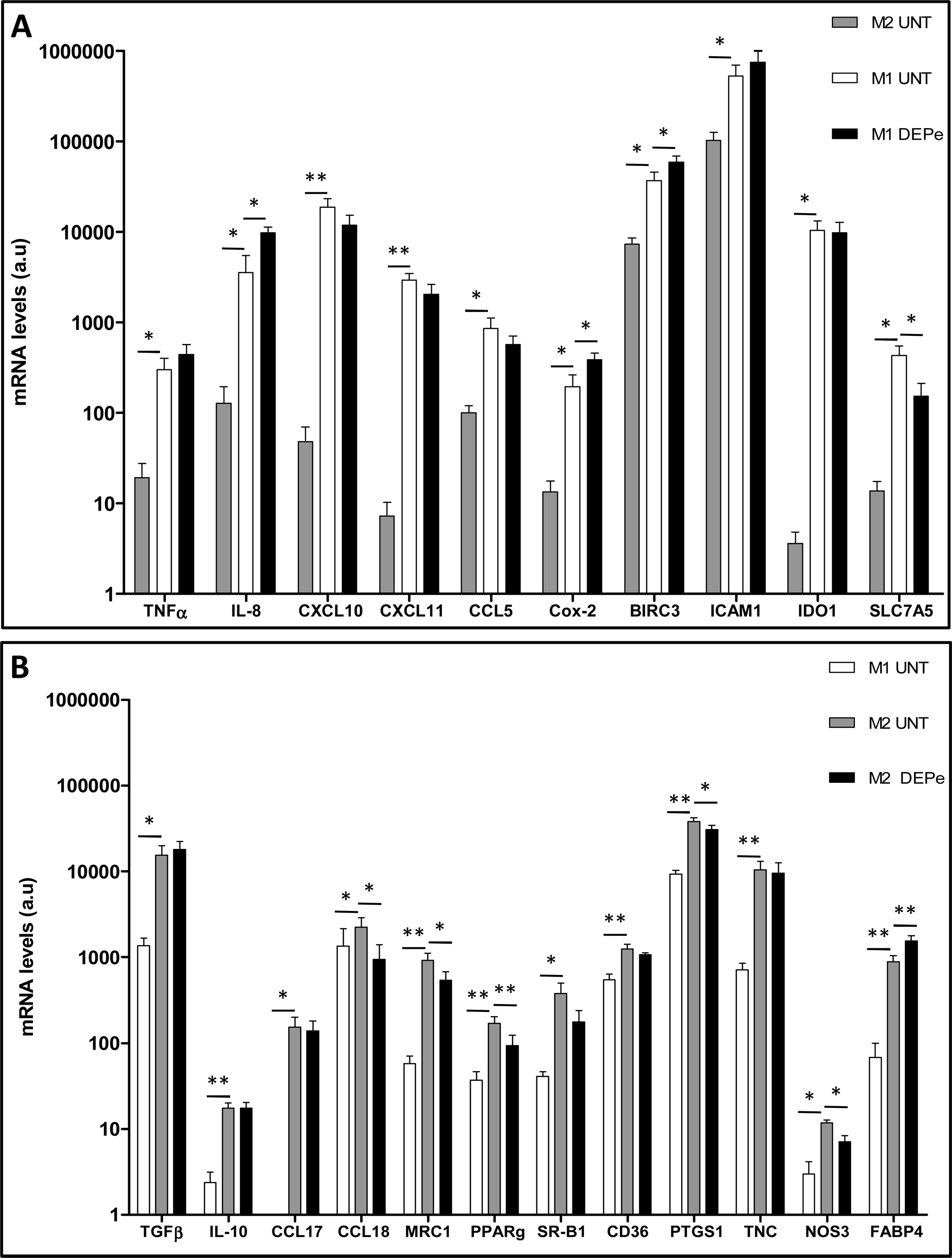

Supplement: S3 Fig — Six-day cultured M-CSF MΦ were activated with IFNγ or with IL-4 to obtain M1 and M2 MΦ respectively, in the presence of 10 μg/ml DEPe or DMSO (UNT) during 24 h. Cells were harvested and after total RNA isolation, mRNA levels were determined by RT-qPCR assays. Quantification of the steady-state target mRNA levels was calculated after normalization of the total amount of cDNA tested to an 18S RNA endogenous reference, using the 2-Ct method. This allowed to get a value of expression for each gene specific of M1 (A) or M2 (B) activation, comparatively to the 18S RNA amount found in RT-qPCR sample, which is presumed to remain constant between the different samples and which was arbitrarily set at 107 units (a.u) (Moreau et al., 2011). Data are the means ± SEM of at least 4 independent experiments. *p<0.05, **p<0.01. (TIF) [file pone.0116560.s003.tif]

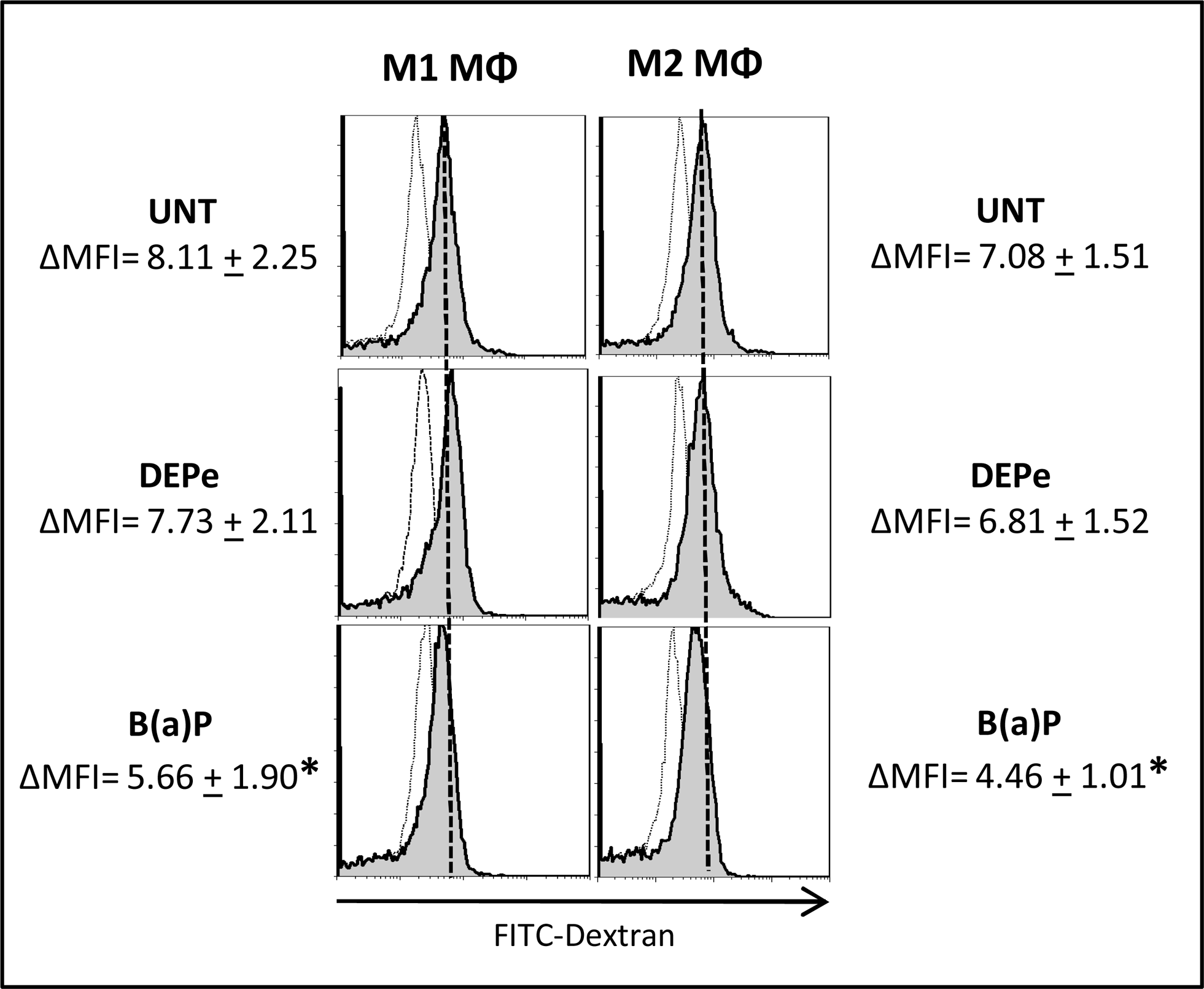

Supplement: S4 Fig — Six-day cultured M-CSF MΦ were activated with IFNγ or with IL-4 to obtain M1 and M2 MΦ, respectively, in the presence of 10 μg/ml DEPe, 2 μM B(a)P or DMSO (UNT) during 24 h. MΦ were incubated with FITC-dextran at 4°C (negative control) or 37°C to measure endocytosis. Cellular uptakes of FITC-dextran, determined by flow cytometry, are expressed as ∆MFI (∆MFI = MFI 37°C—MFI 4°C) and are the means of 7 independent experiment. * p<0.05 when compared with untreated MΦ. Ns: not significant. (TIF) [file pone.0116560.s004.tif]

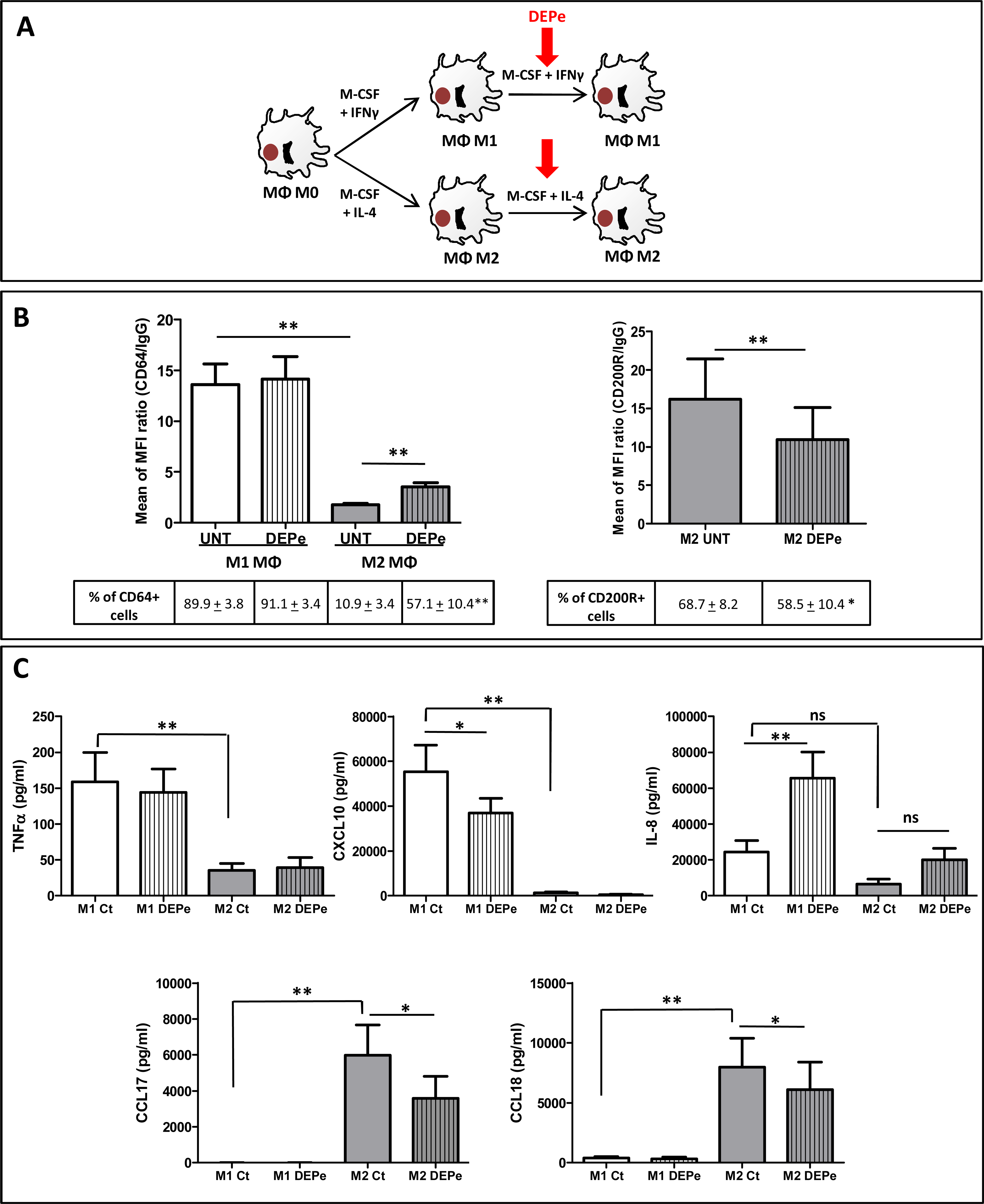

Supplement: S5 Fig — (A) Six-day cultured M-CSF MΦ were activated during 24 h with IFNγ or with IL-4 to obtain M1 and M2 MΦ, respectively. MΦ were then exposed to 10 μg/ml DEPe or to DMSO (UNT) for additional 24 h. (B) Supernatants were collected and cells were then stained with conjugated mAbs directed against the surface markers CD64 and CD200R to be analyzed by flow cytometry. Histograms represent the means of fluorescence intensity (MFI) ratio ± SEM of 7 independent experiments; **p<0.01. (C) Cytokine and chemokine levels in culture medium were determined by ELISA. Data expressed in pg/ml are the means ± SEM of 5 independent experiments. *p<0.05, **p<0.01, ns: not significant. (TIF) [file pone.0116560.s005.tif]
